# Supplementary material for: Oncological Treatment Adverse Reaction Prediction: Development and Initial Validation of a Pharmacogenetic Model in Non-Small-Cell Lung Cancer Patients
Source: Genes (Basel). 2025 Feb 24;16(3):265. doi: 10.3390/genes16030265 (PMC11942520; doi:10.3390/genes16030265)

Gene Ontology (GO) enrichment analysis “Biological Process” - Cluster 0

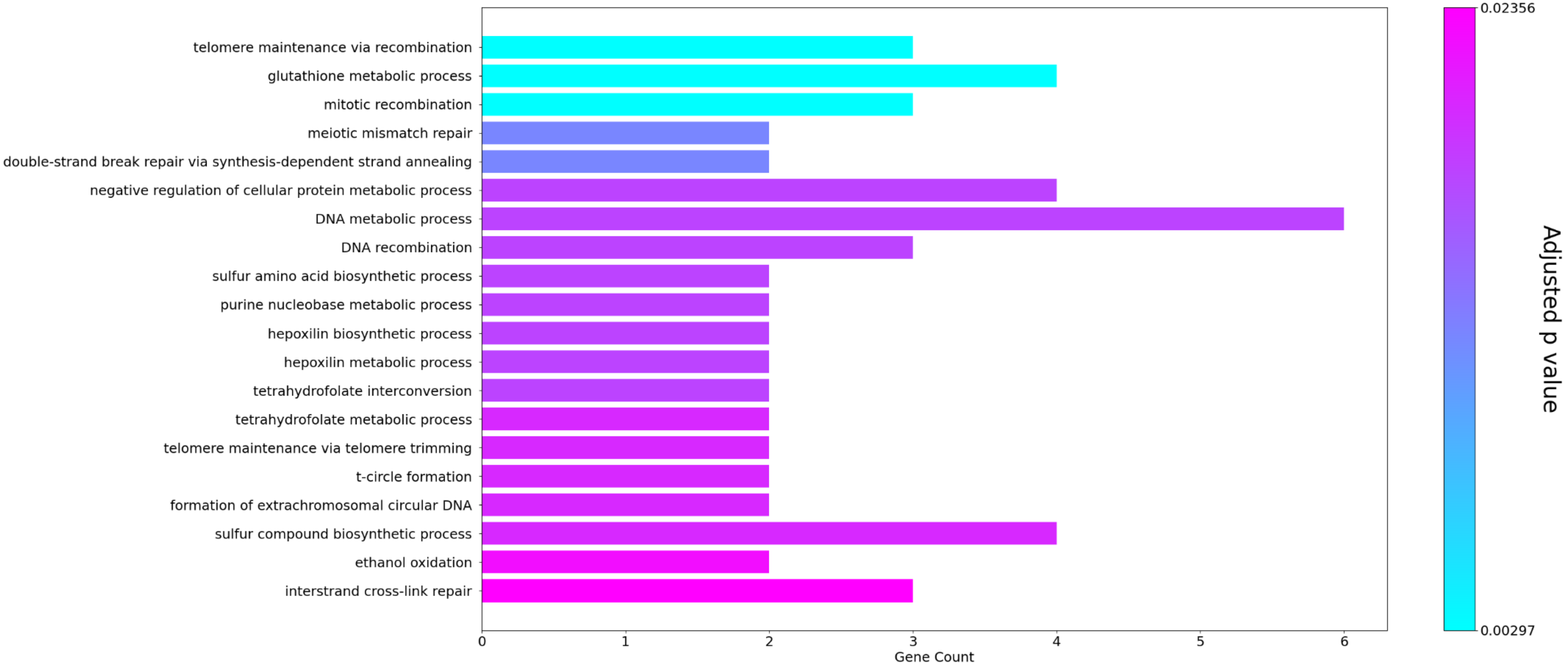

Gene Ontology (GO) enrichment analysis “Biological Process” - Cluster 1

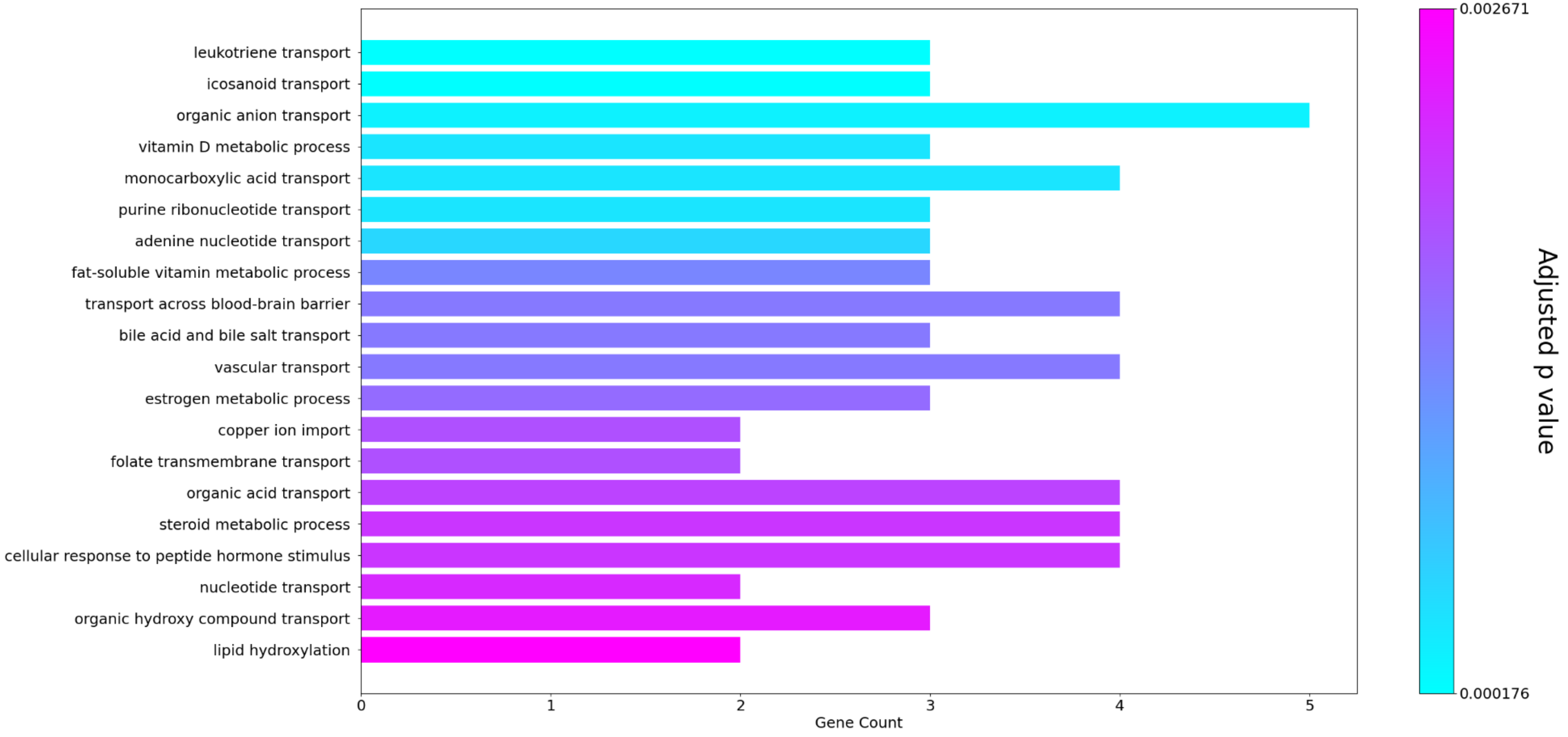

Gene Ontology (GO) enrichment analysis “Biological Process” - Cluster 2

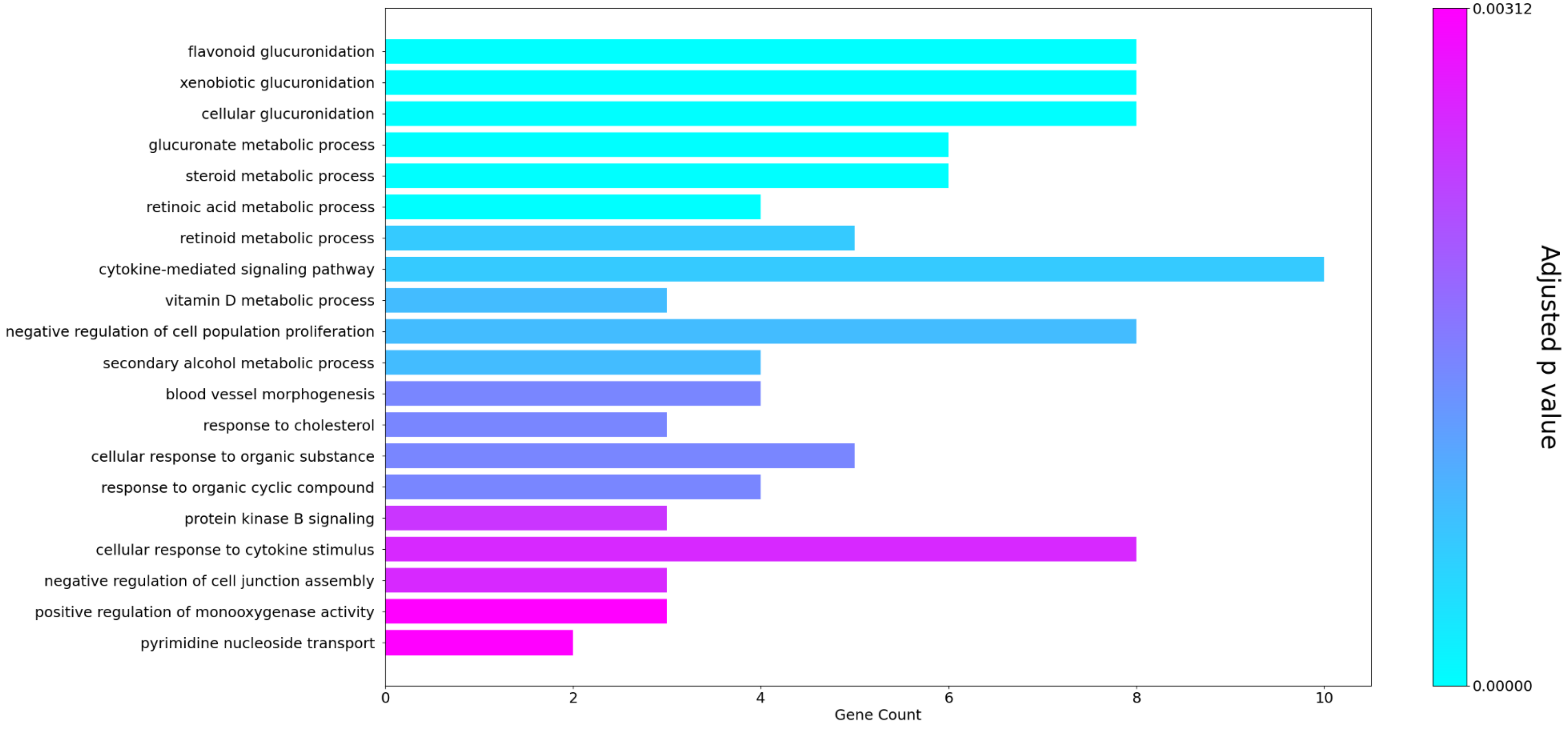

Gene Ontology (GO) enrichment analysis “Biological Process” - Cluster 3

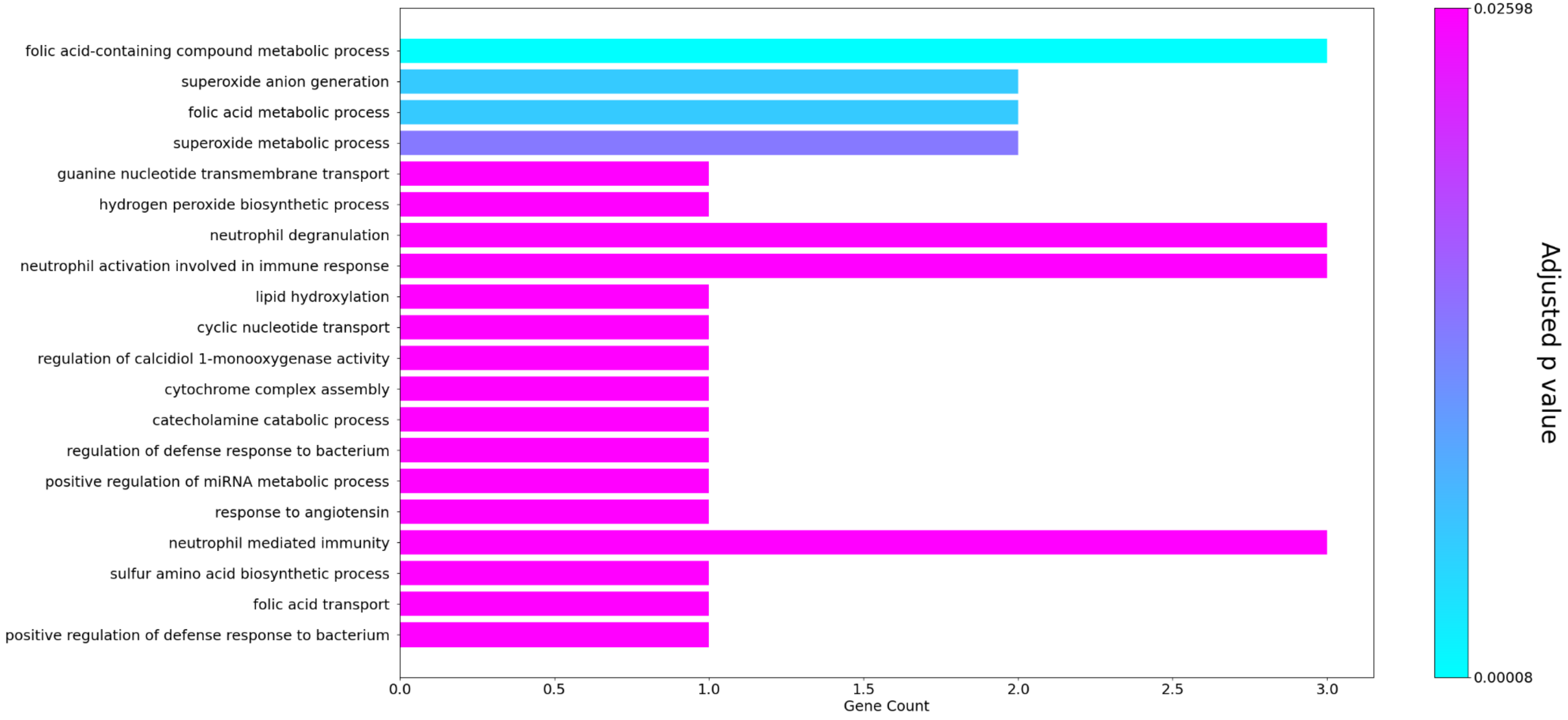

Gene Ontology (GO) enrichment analysis “Biological Process” - Cluster 4

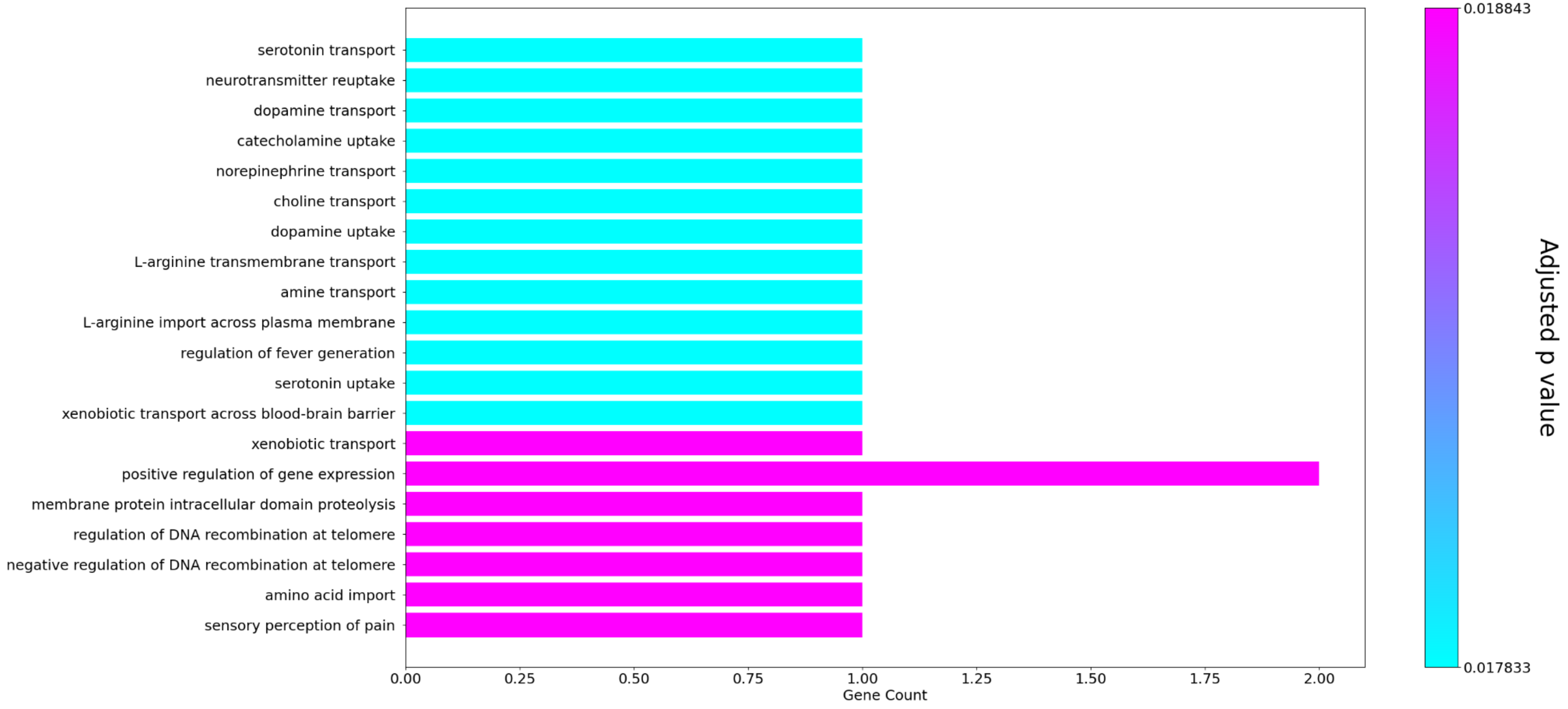

Gene Ontology (GO) enrichment analysis “Molecular Function” - Cluster 0

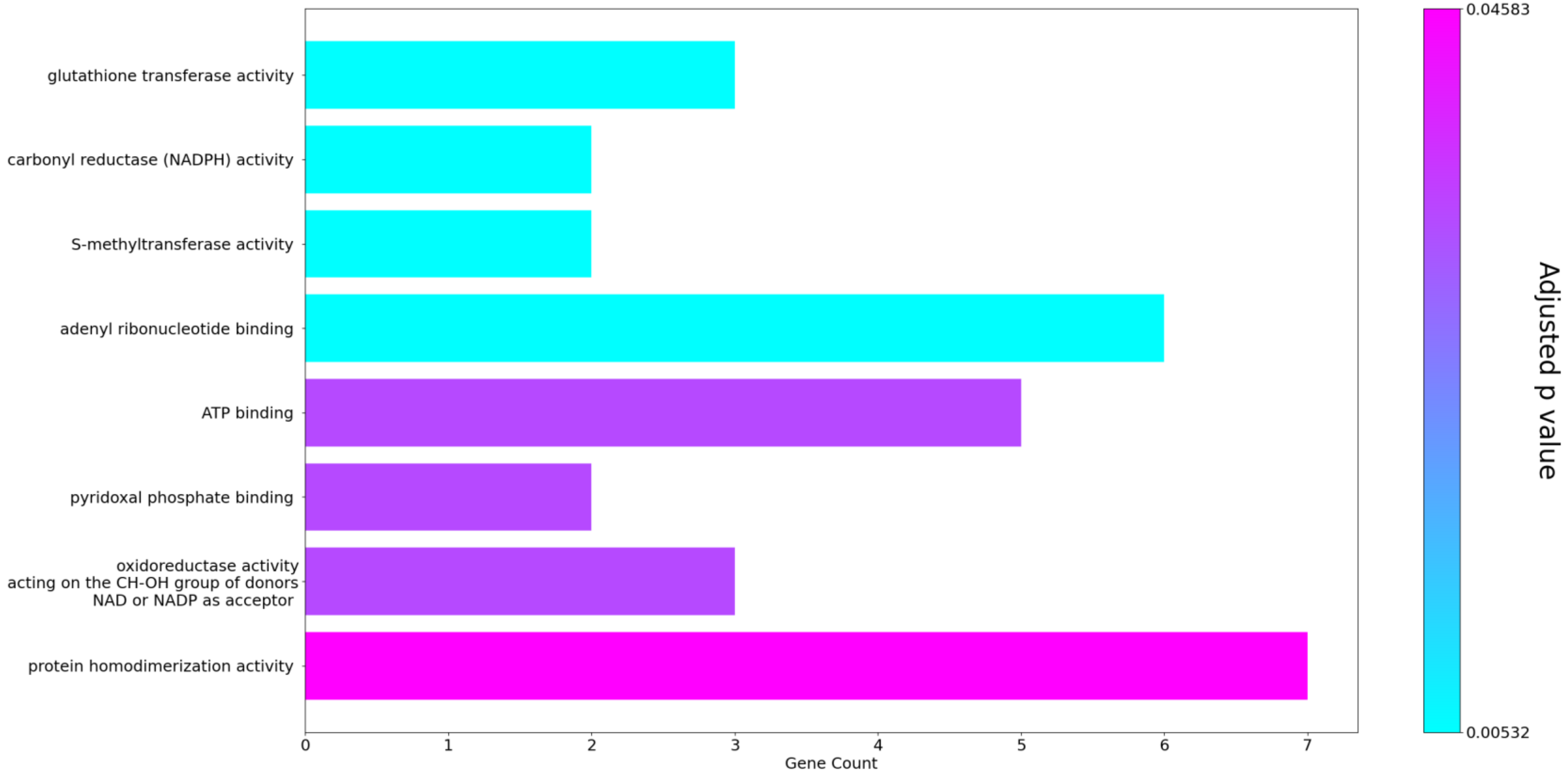

Gene Ontology (GO) enrichment analysis "Molecular Function" - Cluster 1

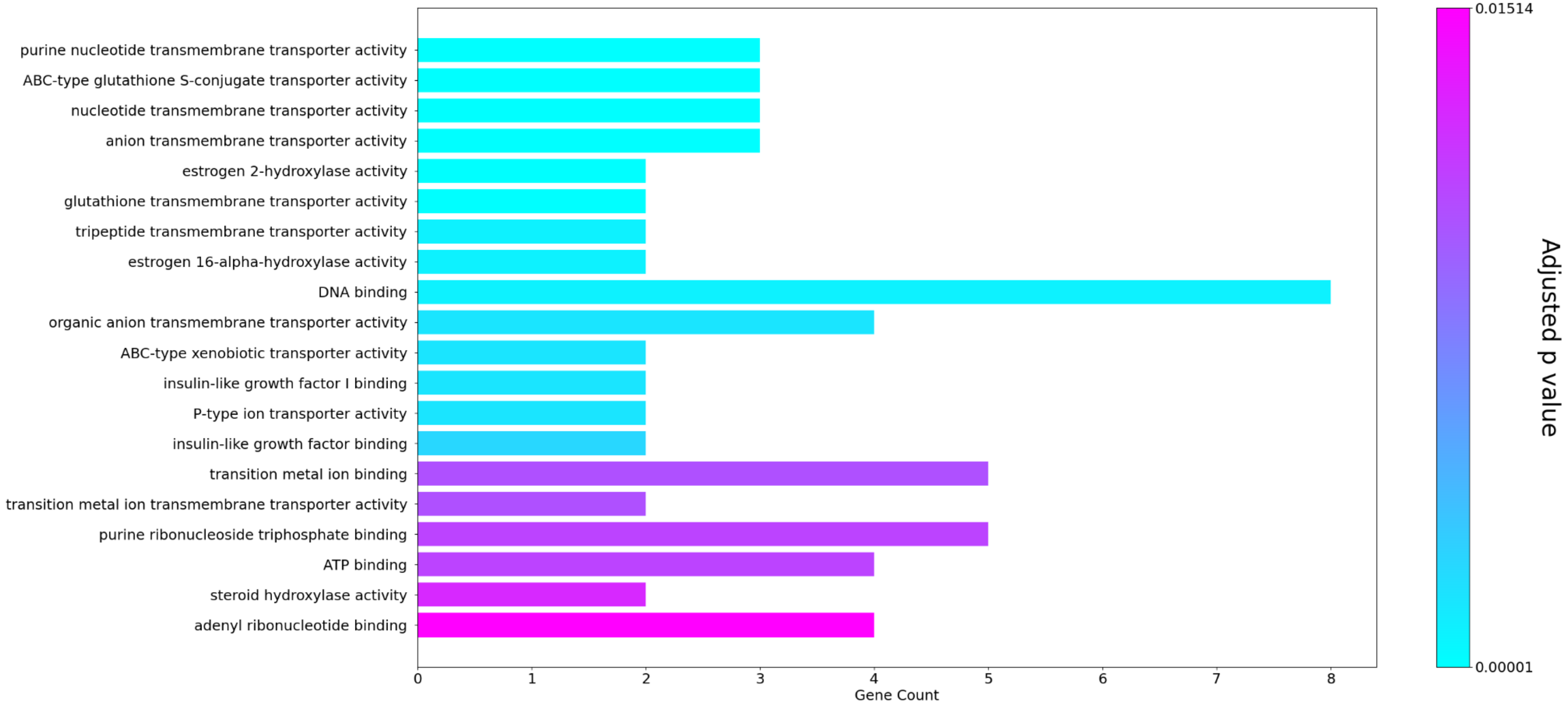

Gene Ontology (GO) enrichment analysis “Molecular Function” - Cluster 2

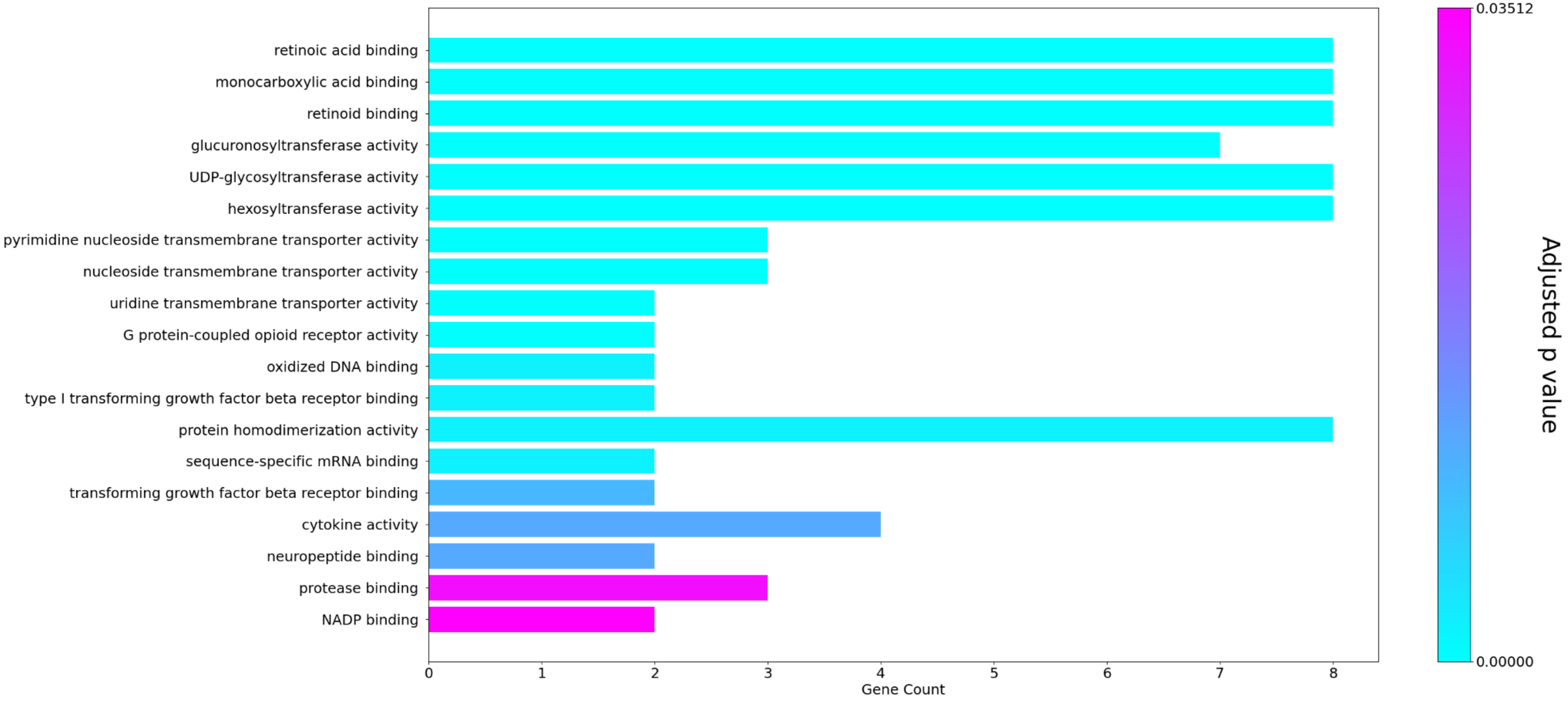

Gene Ontology (GO) enrichment analysis "Molecular Function" - Cluster 3

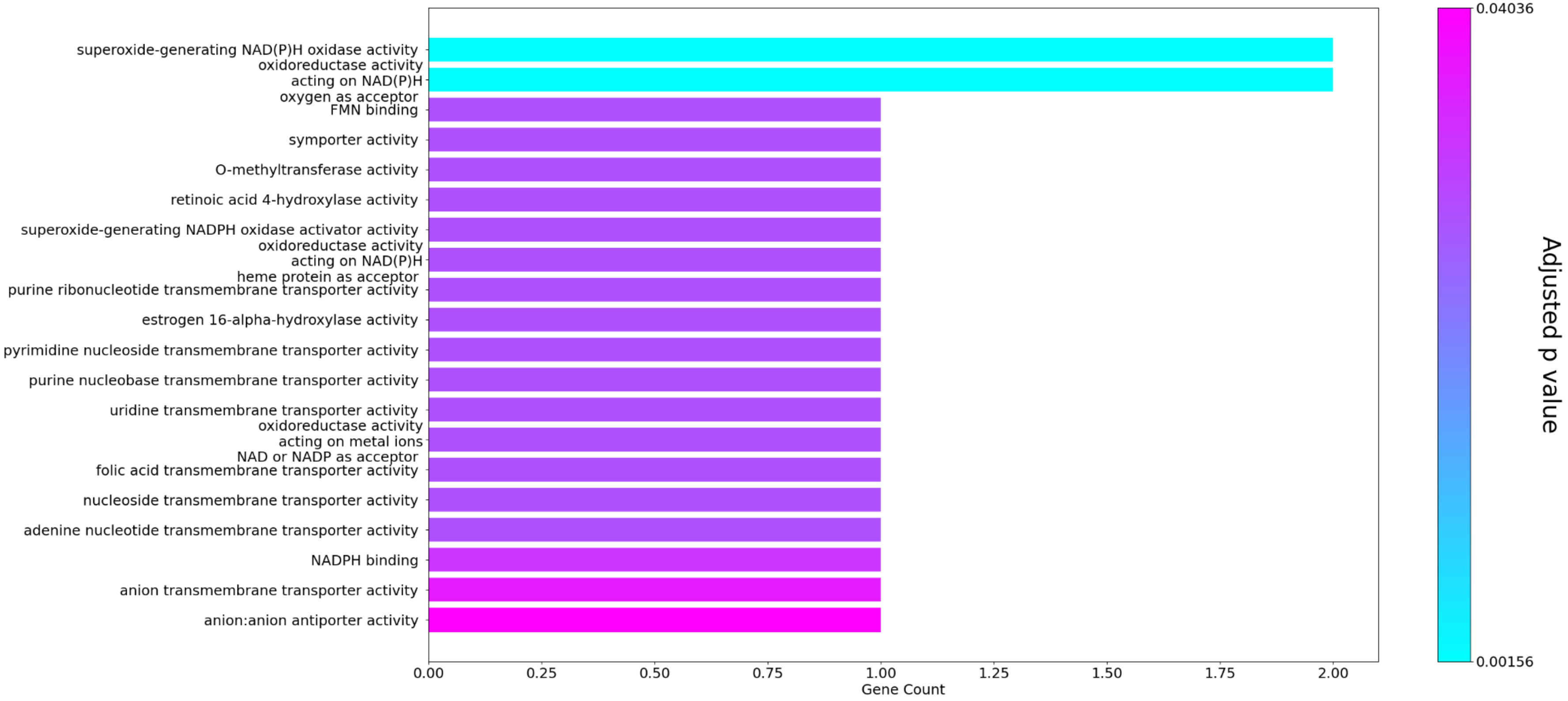

Gene Ontology (GO) enrichment analysis “Molecular Function” - Cluster 4

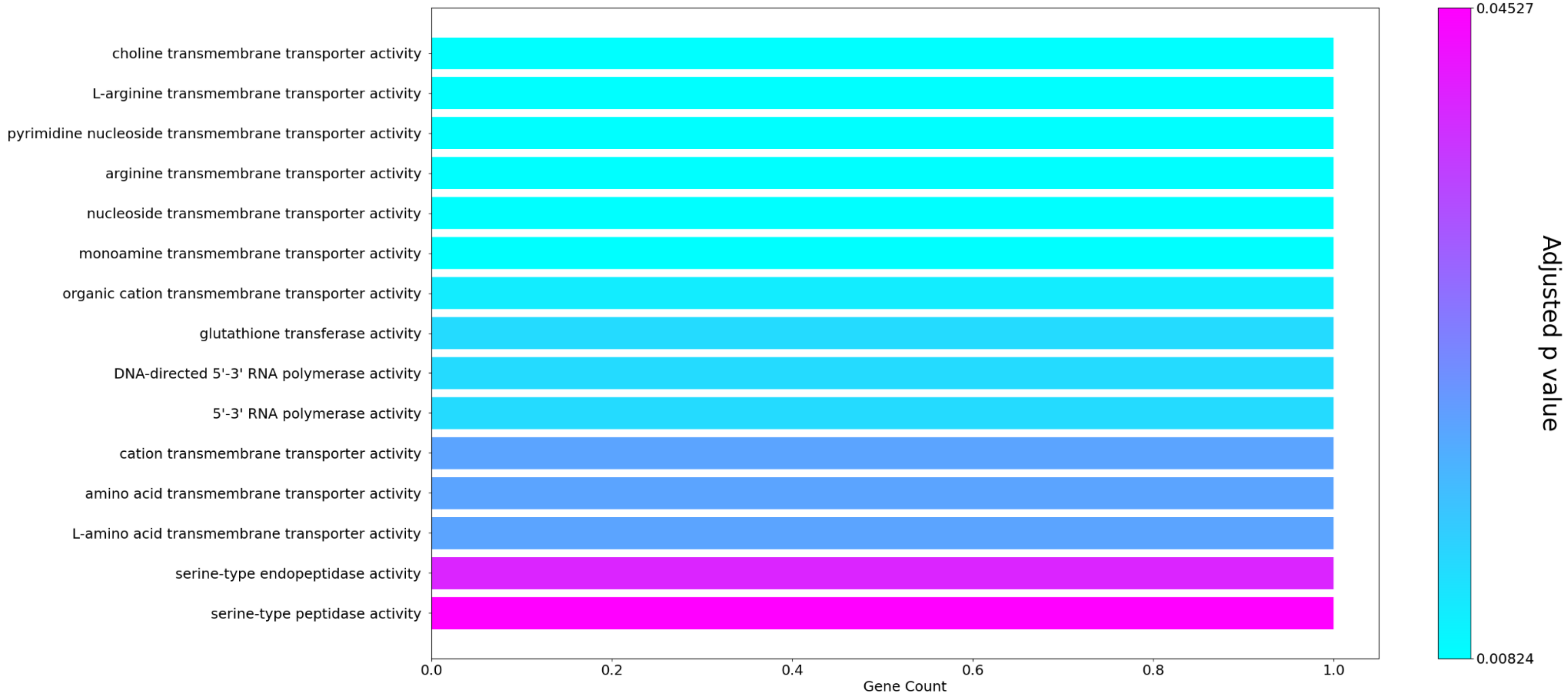

Supplement: Supplementary file 1 [file genes-16-00265-s001.zip › Supplementary Figure 2 .pdf]
