# Supplementary figures and images for: Oncological Treatment Adverse Reaction Prediction: Development and Initial Validation of a Pharmacogenetic Model in Non-Small-Cell Lung Cancer Patients
Source: Genes (Basel). 2025 Feb 24;16(3):265. doi: 10.3390/genes16030265 (PMC11942520; doi:10.3390/genes16030265)

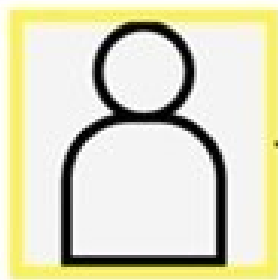

P1

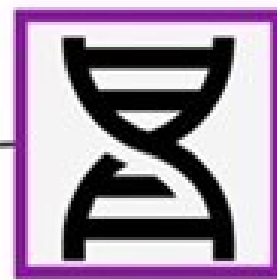

M1

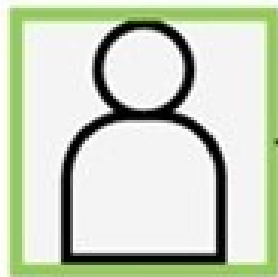

P2

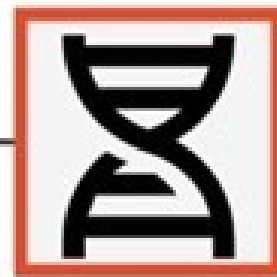

M2

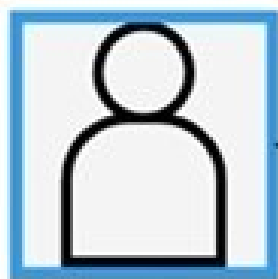

P3

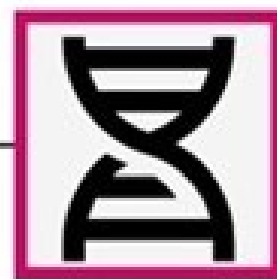

M3

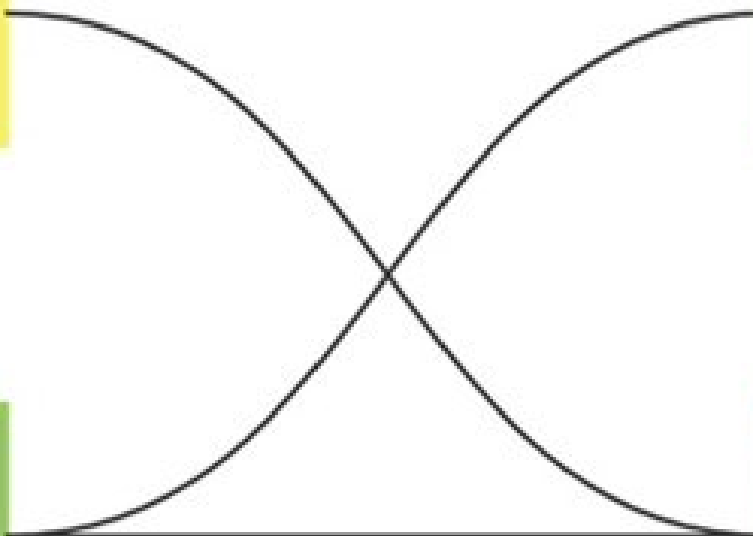

Supplement: Supplementary file 1 [file genes-16-00265-s001.zip › Supplementary Figure 1.pdf]

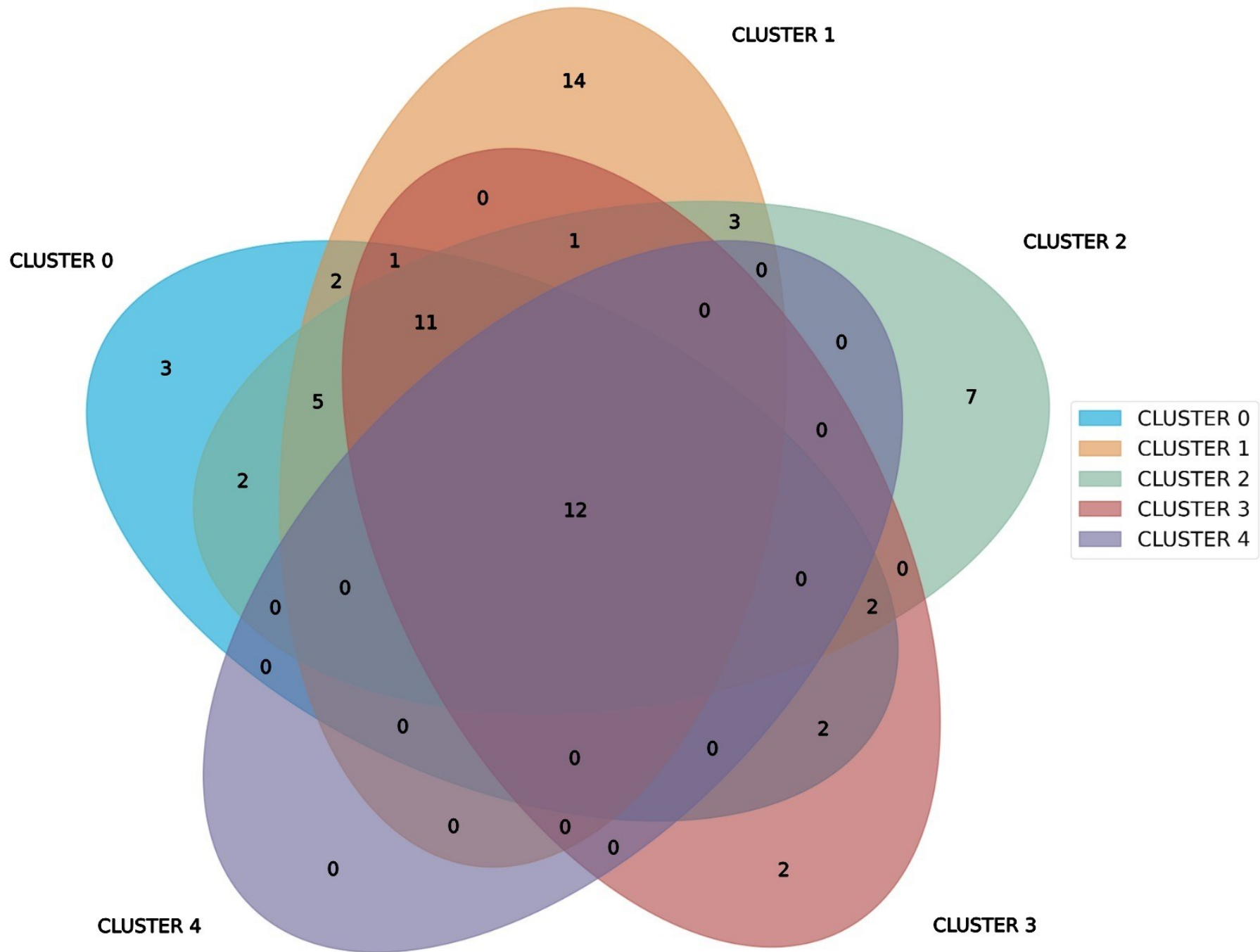

Supplement: Supplementary file 1 [file genes-16-00265-s001.zip › Supplementary Figure 3.pdf]

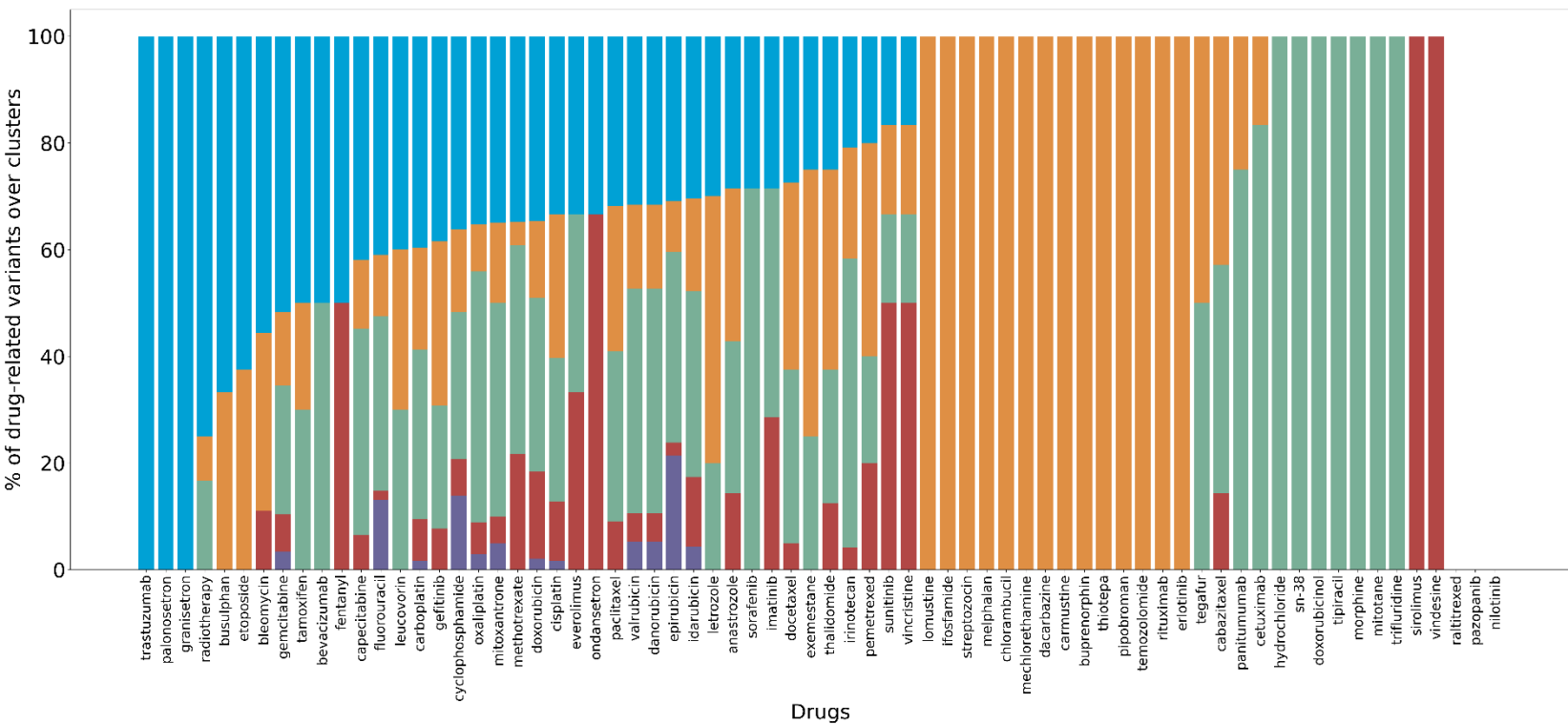

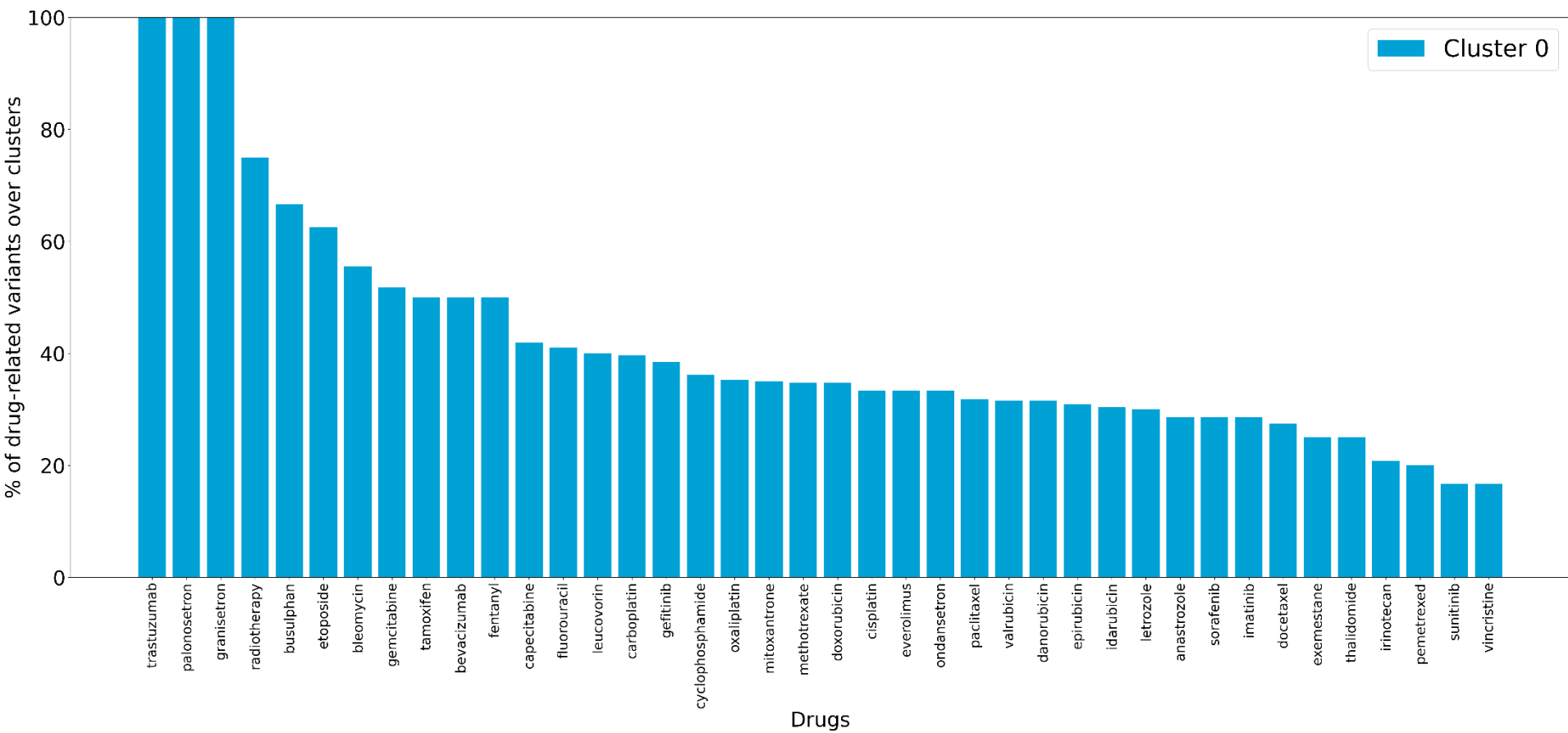

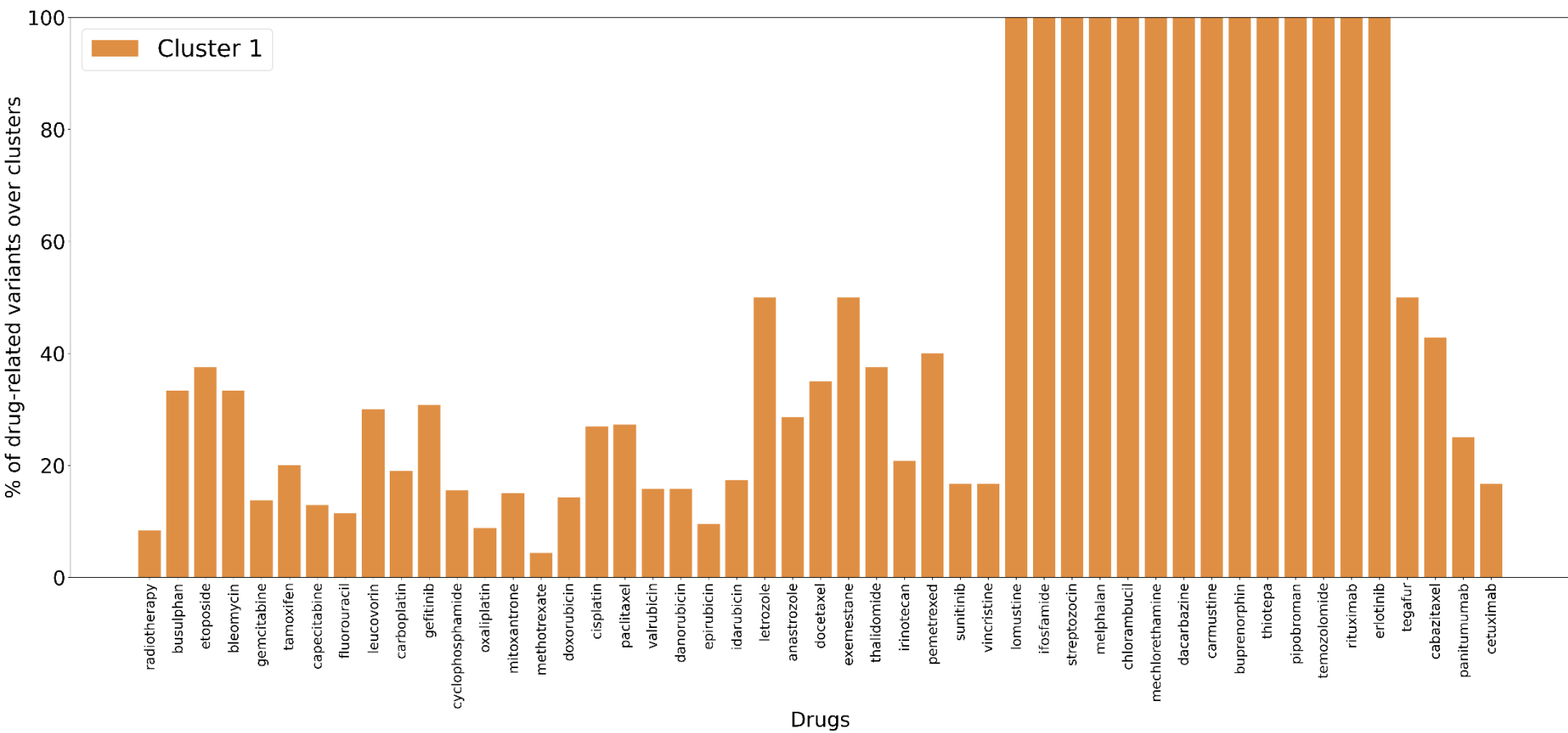

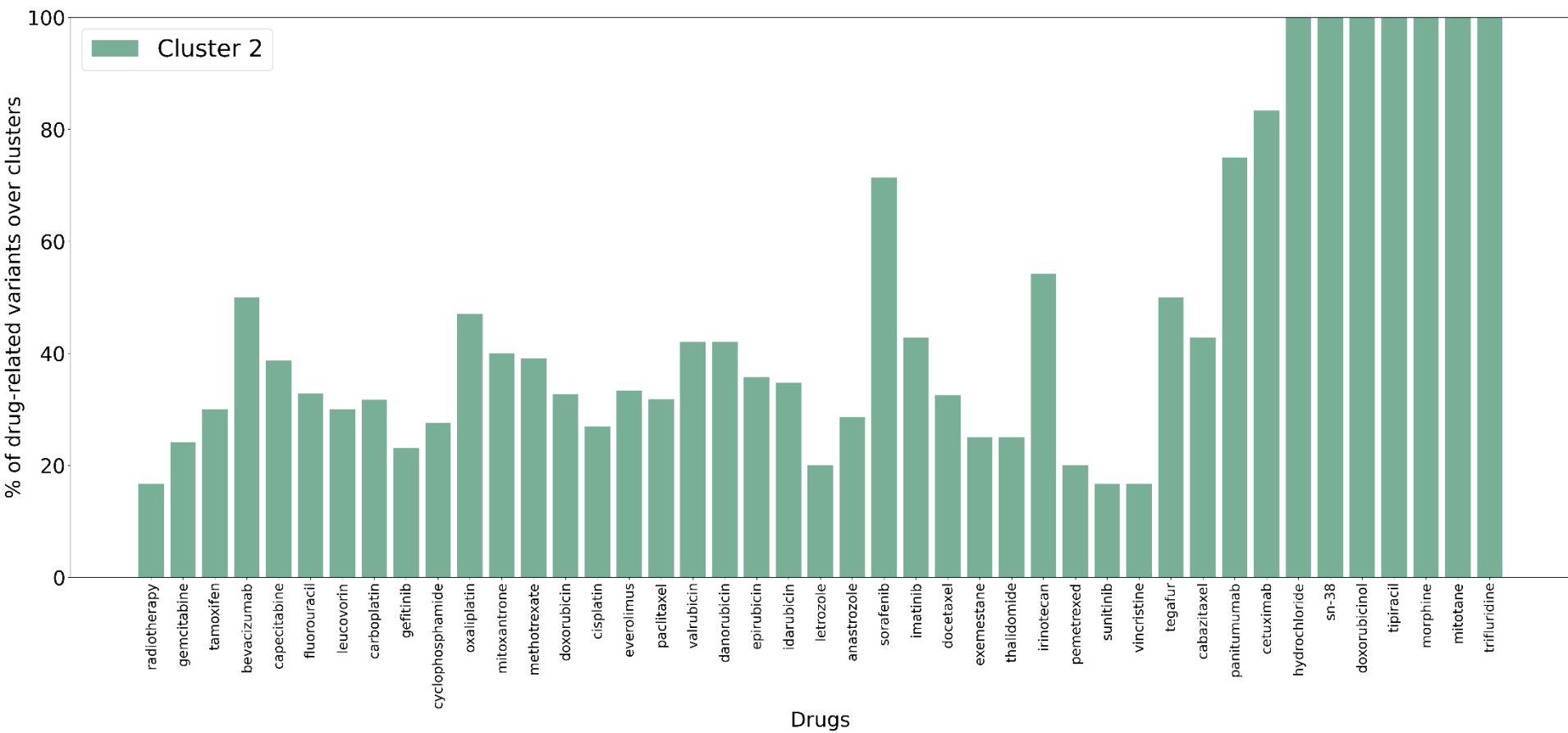

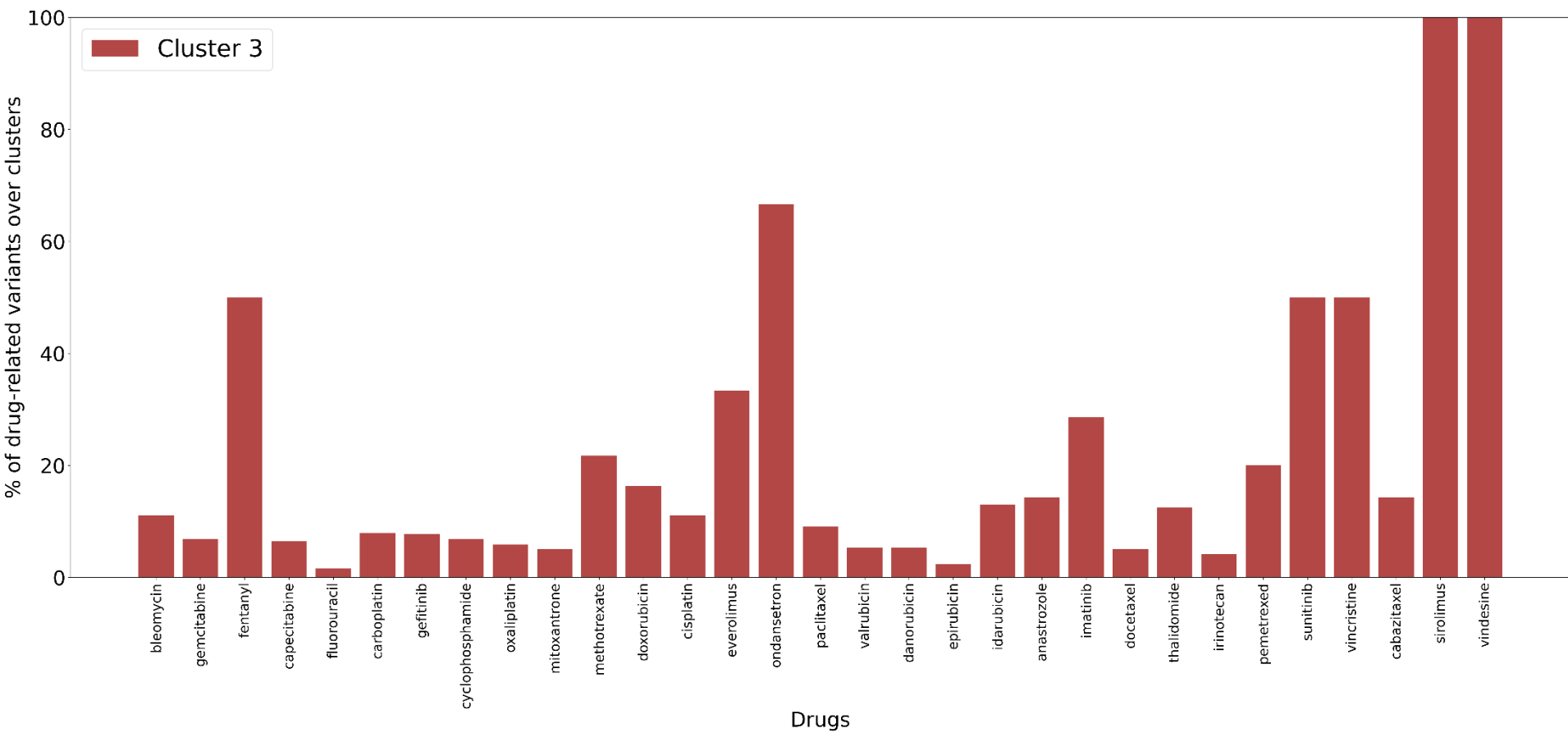

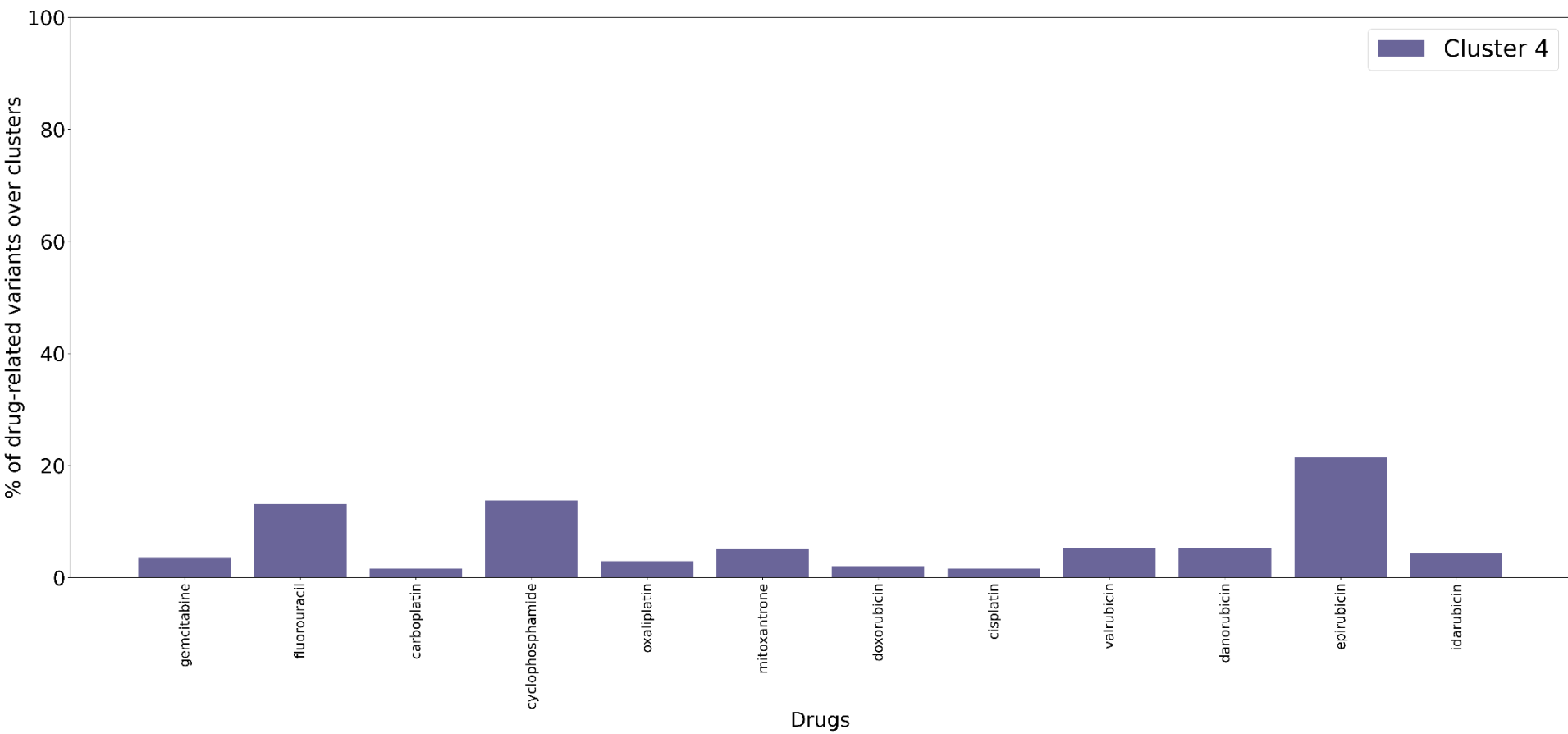

Supplement: Supplementary file 1 [file genes-16-00265-s001.zip › Supplementary Figure 4.pdf]
